# Ceragenin CSA-13 displays high antibacterial efficiency in a mouse model of urinary tract infection

**DOI:** 10.1038/s41598-022-23281-y

**Published:** 2022-11-10

**Authors:** Urszula Wnorowska, Ewelina Piktel, Piotr Deptuła, Tomasz Wollny, Grzegorz Król, Katarzyna Głuszek, Bonita Durnaś, Katarzyna Pogoda, Paul B. Savage, Robert Bucki

**Affiliations:** 1 Department of Medical Microbiology and Nanobiomedical Engineering, Medical University of Białystok, Mickiewicza 2C, 15-222 Białystok, Poland; 2 Independent Laboratory of Nanomedicine, Medical University of Białystok, Mickiewicza 2B, 15-222 Białystok, Poland; 3 Holy Cross Oncology Center of Kielce, Artwińskiego 3, 25-734 Kielce, Poland; 4 Institute of Medical Science, Collegium Medicum, Jan Kochanowski University of Kielce, IX Wieków Kielc 19A, 25-317 Kielce, Poland; 5 Institute of Medical Science, Collegium Medicum, Jan Kochanowski University of Kielce, 25-001 Kielce, Poland; 6 Institute of Nuclear Physics Polish Academy of Sciences, 31-342 Kraków, Poland; 7 Department of Chemistry and Biochemistry, Brigham Young University, Provo, UT 84602 USA

**Keywords:** Antimicrobial resistance, Bacteriology

## Abstract

Ceragenins (CSAs) are synthetic, lipid-based molecules that display activities of natural antimicrobial peptides. Previous studies demonstrated their high in vitro activity against pathogens causing urinary tract infections (UTIs), but their efficiency in vivo was not explored to date. In this study, we aimed to investigate the bactericidal efficiency of ceragenins against *E. coli* (Xen14 and clinical UPEC strains) isolates both in vitro and in vivo, as well to explore CSA-13 biodistribution and ability to modulate nanomechanical alterations of infected tissues using animal model of UTI. CSA-44, CSA-131 and particularly CSA-13 displayed potent bactericidal effect against tested *E. coli* strains, and this effect was mediated by induction of oxidative stress. Biodistribution studies indicated that CSA-13 accumulates in kidneys and liver and is eliminated with urine and bile acid. We also observed that ceragenin CSA-13 reverses infection-induced alterations in mechanical properties of mouse bladders tissue, which confirms the preventive role of CSA-13 against bacteria-induced tissue damage and potentially promote the restoration of microenvironment with biophysical features unfavorable for bacterial growth and spreading. These data justify the further work on employment of CSA-13 in the treatment of urinary tract infections.

## Introduction

Urinary tract infections (UTIs) are defined as infections of the urinary system, affecting either the lower urinary tract or the lower and upper urinary tracts simultaneously, ranging from cystitis (bladder infections) to pyelonephritis (kidney infections)^1,2^. Importantly, UTIs are associated with an increased risk of numerous life-threating and life quality-worsening medical conditions, such as renal scarring in infants, premature birth in pregnant women, or sepsis in the elderly, all of which have significant socioeconomic implications^3^. Urine analysis and urine culture to assess bacteriuria are the common methods to diagnose UTIs. Although urine culture may not be required in the evaluation of outpatients with uncomplicated UTIs, it is required when recurrent UTIs, treatment failures, or complicated UTIs occur^4^. Bacteria, especially those from Enterobacterales family, such as *Escherichia, Klebsiella, Enterobacter* and *Proteus*, are the most common etiological factors of urinary tract infections, but fungi and viruses have also been implicated^2^. Particularly, uropathogenic *E. coli* strains (UPECs) accounts for 80–90% of all UTIs and for this reason *E. coli* strains are used commonly as a model pathogen to research UTI pathogenesis. *E. coli* adhesins enable ascending colonization by allowing pathogens to attach to particular receptors produced by the uroepithelial lining of the urinary system, therefore avoiding removal by urine flow. The dynamically fluctuating shear stress associated with urine flow in the urinary system greatly modulates this phase of pathogenesis^5^. Most UTIs are caused by bacteria originally colonizing gut that sequentially colonize the perineum and then ascend through the urethra^6^. Among the adhesins, the cystitis-associated (type 1) and pyelonephritis-associated (P) pili, encoded by the fim or pap operons, are the most fully described^5^. Over 90% of UPEC strains generate type 1 pili that facilitate mannose-specific adhesion to the bladder epithelium by binding to mannosyl residues through the lectin domain of FimH. Members of the Dr/Afa family, the third most prevalent group of UPEC adhesins, are frequently related with cystitis in children, pyelonephritis, and recurrent treatment-resistant UTIs in young and pregnant women^5^.

Antibiotics are widely used to treat symptomatic UTIs; however, these therapies may change the normal microbiota of the vaginal and gastrointestinal tract over time, resulting in development of multidrug-resistant microorganisms. The presence of niches that have been vacated by the altered microbiota results directly in the increased risk of multidrug-resistant uropathogens colonization, which in light of the approaching end of the “golden age” of antibiotics, forces the development of more rationally planned and alternative therapies. Endogenous antimicrobial peptides (AMPs) have been studied extensively as potential new therapeutic agents for the treatment of drug-resistant microbial infections, including those associated with urinary system^7–9^. Nevertheless, in the light of stability and low in vivo effectiveness^10^, a novel and effective antibiotic class, termed ceragenins (CSA), was developed as synthetic mimics of endogenous AMPs^11^. Ceragenins have been thoroughly investigated in a broad spectrum of in vitro and in vivo models, presenting potent antimicrobial activity against drug-resistant bacteria, fungal pathogens, bacterial spores, viruses and protozoa^12–16^. The foundation of the mechanisms of such activity is membrane permeabilization and generation of excessive oxidative stress in microbial cells^12,13,17^. Our previous studies indicated that ceragenins, particularly CSA-13 and CSA-131, being the representatives of the first and the second generation of CSAs, respectively, are also highly effective against *E. coli* strains isolated from the patients diagnosed with urinary tract infection, and they act against extra- and intracellular bacteria synergistically with host defense molecules, such as human cathelicidin LL-37^7^. Nevertheless, these promising results had not yet been confirmed using an in vivo model, in which lies the novelty of this study. The validity of such approach is also confirmed by our previous report, indicating that intravenously administrated CSA-13 is excreted in the urine^18^, which would be highly favorable for UTI treatment.

Atomic force microscopy (AFM), as a novel, exciting nano-tool, is one of the more recent analytic methods for evaluating anti-inflammatory drugs utilized as both an instrument for nanoscale cell imaging and a force sensor for measuring the shear adhesion force between a cell and its substrate. The evaluation of elasticity modulus (Young's modulus) can be used to understand the mechanical foundation of disease development by determining the local cell or tissue stiffness in detecting morphological and precise ultrastructural changes of the cell membrane at the nanoscale^19^. Atomic force microscopy makes it possible to assess the immunomodulatory effects of the compounds under study, including through changes in the size and roughness of cell membrane particles, which is related to an indicator of inflammatory responses^20^. Recent research indicates that alterations in the nanomechanical characteristics of cells represent the transformation of cell physiology through the processes of molecule presentation and identification, signal detection, and enhanced production of surface molecules or cell activation. Using the relative value of Young's modulus^21^, the magnitude of this phenomena may be quantified. Importantly, the opposite of stimuli-induced cellular responses may serve as a signal of the biological activity of some medications^22^. Previously, AFM was utilized successfully as an analytical tool to detect LPS-induced inflammatory responses in macrophages and monocytic cell cultures^20,22^, to analyze mechanisms of pathophysiological neutrophil mechanics in endotoxemia-related inflammatory conditions^23^, and to evaluate the reduction of stiffness-dependent exacerbation of inflammatory processes^24,25^.

In this study, to test the in vivo efficiency of ceragenins in a urinary tract infection model, mice with induced UTIs were treated with CSA-13, followed by urine collection and microbial culture, live scanning of mice using near-infrared camera to investigate the biodistribution of administrated compound and further analysis of bladder tissues collected from sacrificed animals using histopathology staining and atomic force microscope (AFM), allowing to understand the mechanical foundation of treatment progress.

## Results

### Ceragenins display potent antimicrobial activity against E. coli Xen14, which is conditioned by the induction of reactive oxygen species

**Figure 1:**
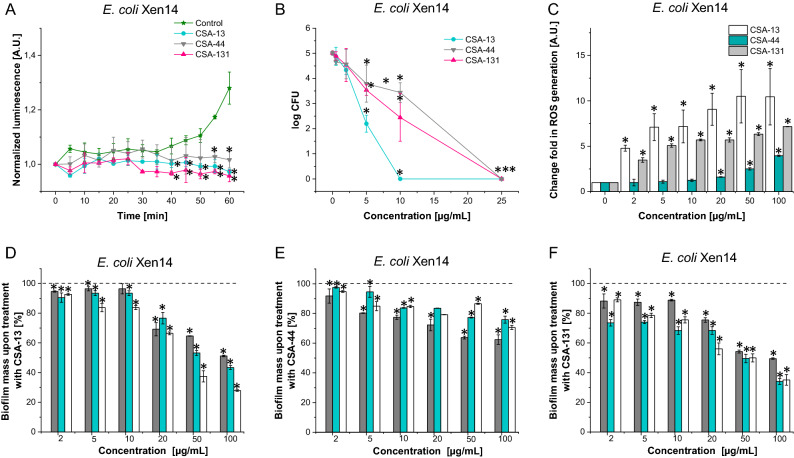
Antibacterial activity of ceragenins (CSA-13, CSA-44 and CSA-131) against *E. coli* Xen14. Decrease of bacteria-derived luminescence signal upon 1 h treatment with tested ceragenins. Results are presented as mean ± SD from 3 replicates (**A**). Decrease of survival of *E. coli* Xen 14 planktonic bacteria when exposed to ceragenin CSA-13, CSA-44 and CSA-131 evaluated using the “killing assay” method. Results are presented as mean ± SD from 3 replicates (**B**). Induction of reactive oxygen species (ROS) generation by *E. coli* Xen14 was evaluated by DFCH-DA fluorometric assay. Formation of ROS upon treatment with CSA-13, CSA-44, and CSA-131 at a concentration of 1–100 μg/mL was presented. Results are presented as mean ± SD from 3 replicates; *indicates statistical significance ≤ 0.05, ** ≤ 0.01, and *** ≤ 0.001 (**C**). Anti-biofilm properties of CSA-13 (**D**), CSA-44 (**E**), CSA-131 (**F**) against *E. coli* Xen14. Ability of ceragenins to prevent the biofilm formation of *E. coli* was measured using crystal violet staining upon 24 (grey bars), 48 (green bars) and 72 h (white bars). Results are presented as mean ± SD from 3 replicates. Dashed horizontal line indicate untreated control (0 µg/mL of ceragenins).

As seen in Table 1, bioluminescence strain *E. coli* Xen 14 and UPEC clinical strains (S1, S2) are sensitive to ceragenins (CSA-13, CSA-44 and CSA-131). The MICs (minimal inhibitory concentrations) and MBCs (minimal bactericidal concentrations) of ceragenins against *E. coli* were from 1 to 4 µg/mL and from 2 to 8 µg/mL, respectively. Importantly, bactericidal activities of CSA-13 were also maintained in different body fluids (Table 2). Results collected in this step confirmed the utility of CSA-13-IRDye 800CW in further stages of our research, since its bactericidal activity was not changed in LB (Luria–Bertani) broth, urine and plasma when compared to unmodified CSA-13 and recorded differences were not higher than well-acceptable error ranges of this method. In the next step, we evaluated the effect of these ceragenins (CSA-13, CSA-44 and CSA-131) against *E. coli* Xen14 strain by monitoring changes in bacterial luminescence signals. Changes in *E. coli* Xen14-derived luminescence signals after treatment with ceragenins during 60 min are presented in Fig. 1A. In contrast to the control sample, for which an increase of luminescence signal resulting from bacteria division and considerable metabolic activity of bacterial cells was observed, the luminescence signal of *E. coli* Xen14 was reduced after 35 min of treatment with ceragenins. We also evaluated the antibacterial activity of these ceragenins with a bacterial killing assay (Fig. 1B). Ceragenin CSA-13 had greater bactericidal activities against *E. coli* Xen14 than CSA-44 and CSA-131; a complete inhibition of bacteria growth at the concentration of 10 µg/mL of CSA-13 was recorded, while for CSA-44 and CSA-131, the comparable killing effect was observed at 25 µg/mL. These results were corroborated by measurement of oxidative stress, through DCFH-DA-derived (2′,7′-Dichlorofluorescin diacetate) signal recording (Fig. 1C). Collected data confirmed our previous results indicating that ceragenin bactericidal activity results primarily from induced oxidative stress^12,26^. A statistically significant, ~ 4-fold increase of ROS (reactive oxygen species) production, compared to the unstimulated control, was observed at 2 µg/mL of CSA-13 and this effect enhanced with the increasing concentration of all ceragenins. Finally, we looked into the possibility of using ceragenins to prevent biofilm formation by *E. coli* Xen14. The most effective in preventing biofilm formation were CSA-13 and CSA-131, as shown in panels D-F of Fig. 1.

**Table 1:** Minimal inhibitory concentration and minimal bactericidal concentrations (MIC/MBC; µg/mL) of CSA-13, CSA-44 and CSA-131 against *Escherichia coli* Xen14 and two clinical strains of uropathogenic *E. coli* (S1 and S2) isolated from patients with urinary tract infections.

	CSA-13	CSA-44	CSA-131
*E. coli* Xen14	2/2	4/8	1/2
*E. coli S*1*	1/2	2/4	1/2
*E. coli S*2*	2/4	2/4	2/4

*Clinical strain.

**Table 2:** Minimal inhibitory concentration and minimal bactericidal concentrations (MIC/MBC; µg/mL) of CSA-13 and CSA-13-IRDye800CW, against *E. coli* Xen14 and two clinical strains of uropathogenic *E. coli* (S1 and S2) isolated from patients with urinary tract infections in LB broth, urine and plasma.

	CSA-13			CSA-13-IRDye 800CW		
	*E. coli* Xen14	*E. coli* S1*	*E. coli* S2*	*E. coli* Xen14	*E. coli* S1*	*E. coli* S2*
LB broth	2/2	1/2	2/4	1/2	2/4	2/4
Urine	2/4	1/2	2/4	4/8	4/8	2/4
Plasma	4/8	2/4	4/8	4/8	4/8	4/8

*Clinical strain.

### CSA-13 at bactericidal concentration displays high biocompatibility and protects bladder cells against bacterial induced death

**Figure 2:**
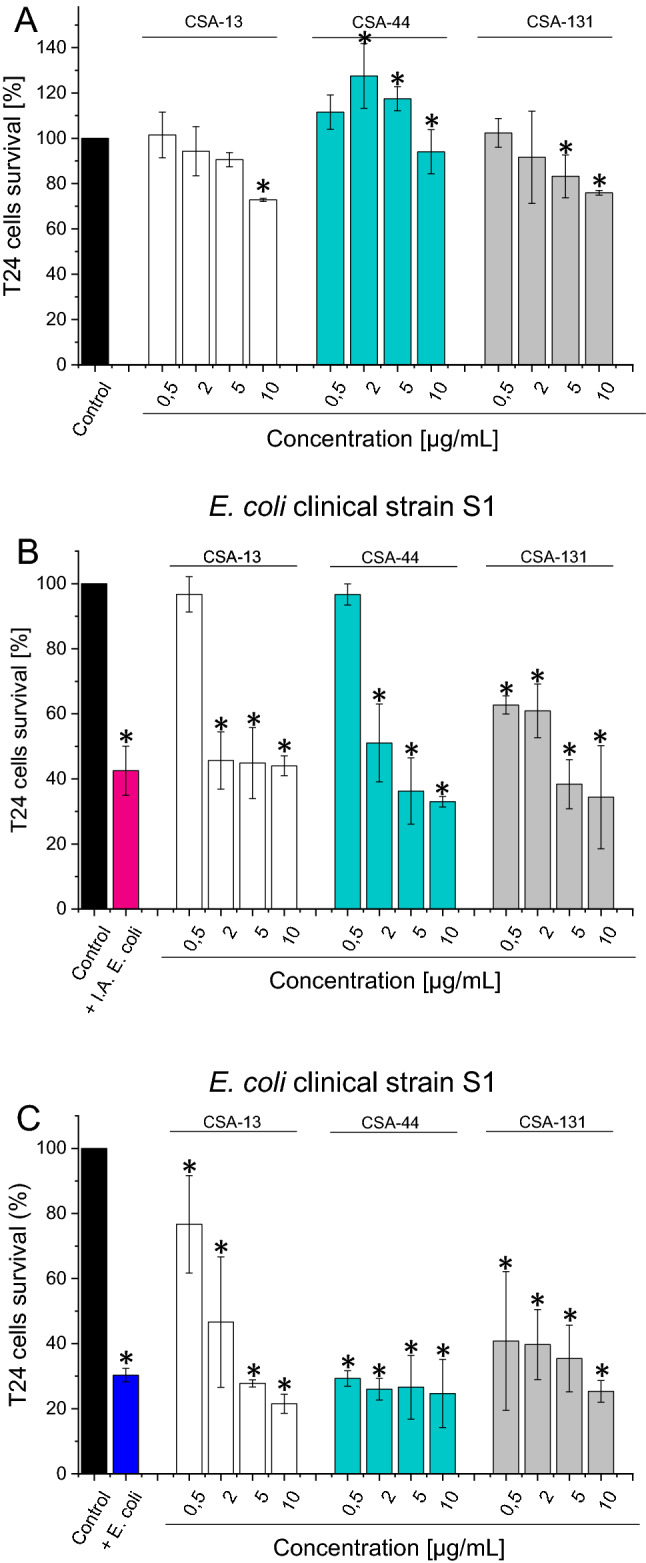
Survival of human urinary bladder cancer cells T24 (HTB-4™) upon incubation with CSA-13, CSA-44 and CSA-131 at doses of 0–10 µg/mL for 24 h (**A**). Cytotoxic effect of cationic lipids against T24 cell line after cells incubation with both heat-inactivated (**B**) and life (**C**) *E. coli*.

To evaluate potential ceragenin toxicity, we performed measurement of cell viability after incubation of human bladder cell line with antibacterial agents at different concentrations (Fig. 2, panel A). Ceragenin CSA-13 affect the survival of T24 (human bladder cancer cell line) cells at concentration range 0.5–10 µg/mL, at limited manner. Notably, significant bactericidal effects of the tested agents were observed at lower, non-cytotoxic levels, highlighting their applicability in the treatment of *E. coli*-caused urinary tract infection. An increase in cytotoxicity towards T24 cells was observed after CSA-131 treatment. At a concentration of 5 µg/mL there was a decrease in cell survival by almost 20%, in contrast to CSA-44, where we did not observe any significant effects on the survival of T24 cells at tested concentrations. In another experimental setting, we investigated whether tested ceragenins are able to protect urinary bladder cells against bacteria-induced cell death. As demonstrated in Fig. 2B and C, culturing of cells in the presence of heat-inactivated or live *E. coli* cells resulted in decreases in cell viability by up to 40% and 30%, respectively. Nevertheless, after 1 h incubation of *E. coli*-infected cells with ceragenins, we observed that the ceragenins, primarily CSA-13, promote the survival of T24 infected cells (Fig. 2B and C) and protected bladder cells from damage caused by bacteria. Collectively, these in vitro results motivated us to carry out further analyzes in an animal model.

### CSA-13 upon intravenous administration is excreted by kidneys and liver

**Figure 3:**
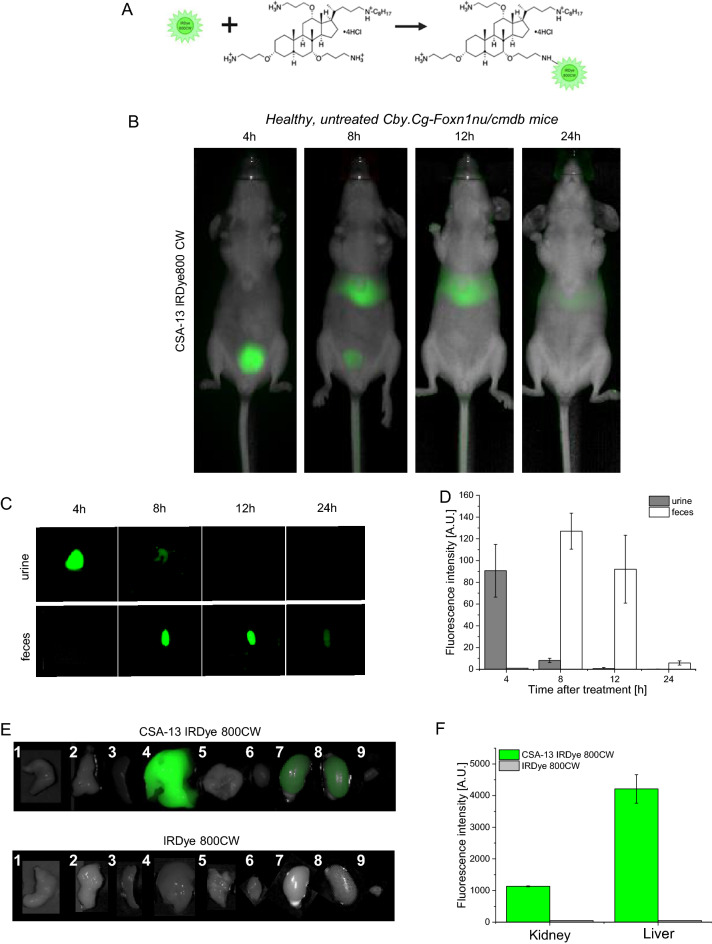
Scheme description of CSA-13 labeling with IRDye 800CW (**A**). Biodistribution of intravenously administrated CSA-13 labeled with IRDye®800CW (CSA-13-IRDye800CW) estimated by fluorescence-based analysis of CSA-13-IRDye800CW-targeted fluorescence signal in healthy, non-infected Cby.Cg-Foxn1nu/cmdb mice (n = 5; group 8) 4, 8, 12 and 24 h post injection of CSA-13 IRDye800CW. Results from representative animals are shown (**B**). Representative scans of urine and feces collected from healthy mice (n = 5; group 8) 4, 8, 12 and 24 h after injection of CSA-13-IRDye800CW (**C**). Presence of labeled CSA-13 in urine and feces was estimated based on fluorescence intensity of collected excreta and presented as mean value ± SEM from all areas of each urine and feces (**D**). Organ uptake of CSA-13-IRDye800CW (group 8) and IRDye800CW (group 9) after 24 h post its administration was estimated based on fluorescence intensity of collected organs (1—stomach, 2—pancreas, 3—spleen, 4—liver, 5—lungs, 6—heart, 7—left kidney, 8—right kidney, 9—bladder) and presented as mean value ± SEM from all areas of each organs (**E**, **F**).

The summary of the animal model design and the overall synthesis process for fluorescently-labeled CSA-13 (CSA-13-IRDye800CW) are shown in Fig. 3. In the first step, biodistribution of healthy, non-infected mice, administrated compound was investigated using real-time bioluminescence imaging (Fig. 3, panel B). The collected scans revealed that 4 h after *i.v.* (intravenous) injection of CSA-13-IRDye800CW into a tail vein of mice, the compound is accumulated mostly in the in the lower abdomen area and close to the bladder. However, 8 and 12 h after injection, the ceragenin-derived signal was also recorded in the liver and possibly material in the bladder. At 24 h post-injection, we observed residual CSA-13-IRDye800CW-derived signal only in the liver. These results were confirmed by analyzing both urine and feces collected from mice after intravenous injection of CSA-13-IRDye800CW. As shown in Fig. 3C, 4 h after intravenous injection of the tested compound, its presence was observed only in the urine of animals. After 8 h, excretion of CSA-13-IRDye800CW with feces was recorded. The fluorescence intensity decreased after the next 12 and 24 h (Fig. 3, panel D). At 24 h post injection, organs were removed from the sacrificed mice, which allowed for the assessment of the accumulation of the tested compounds. As shown in Fig. 3, panels E and F, fluorescence intensity was recorded in kidneys and liver. After 24 h we did not observe liver fluorescence; however, observations made at earlier time points suggest that CSA-13 accumulated in the liver and much less in the kidneys. To confirm that accumulation in the liver and kidney involves the CSA-13-IRDye800CW compound, we also evaluated the biodistribution of the dye itself. At 24 h, the dye was absent in the liver and kidneys.

### CSA-13 eradicates bacteria-causing urinary tract infection in a mouse model

**Figure 4:**
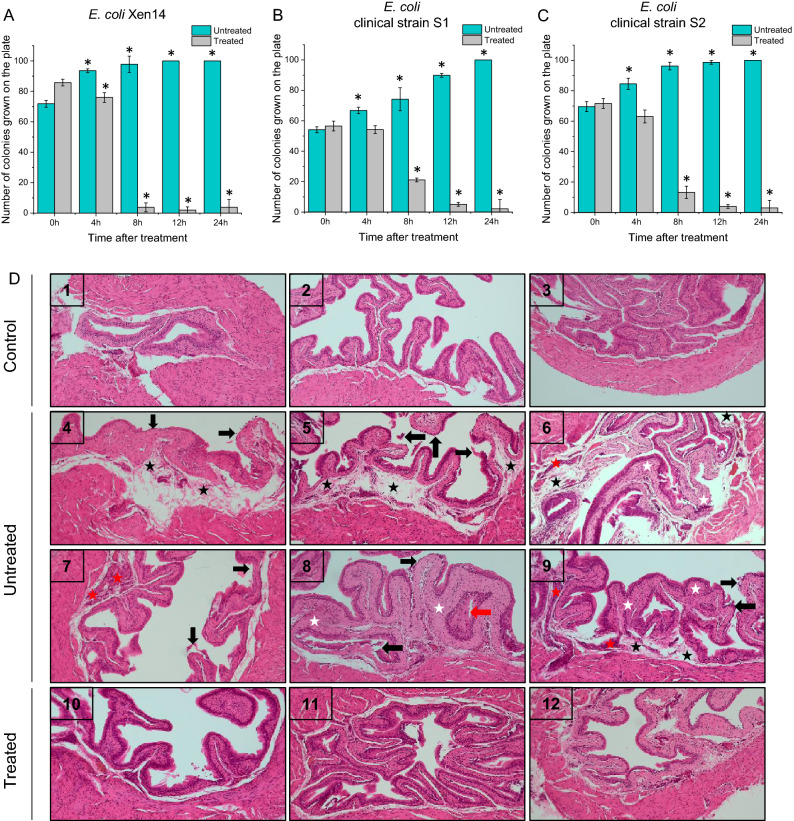
Decrease in *E. coli* Xen14 colonies in urine culture from mice post CSA-13 IRDye800CW treatment (grey columns) when compared to urinary tract infected untreated mice (green columns) (**A**) and decrease in *E. coli* clinical strains (S1, (**B**) and S2 (**C**), colonies in urine culture from mice post CSA-13 treatment (grey columns) when compared to urinary tract infected untreated mice (green columns). Histological analysis of mice bladder tissues: (1–3) normal murine bladder; (4–9) murine bladder infected with *E. coli* Xen14; tissue edema (black star), exfoliation of transitional epithelial cells (black arrow), invasion of inflammatory cells in the mucosa (red star), and bladder mucosa hyperplasia (red arrows) (10–12) Murine bladder infected with *E. coli* Xen14 and treated with CSA-13 (**D**).

At 24 h after bacteria challenge, urine of all animals, both untreated and treated with CSA-13 was collected and cultured to assess the status of bacterial burden and infection development. Importantly, we observed a statistically significant decrease in the number of *E. coli* Xen14 colonies even 4 h after administration of CSA-13-IRDye800CW into the bloodstream of UTI-suffering animals (Fig. 4, panel A). Moreover, 8 h after treatment no significant bacterial growth was detected. In contrast, in the untreated mice considerable bacterial growth was observed at each time point. The same phenomenon was detected when urine was collected from animals inoculated with clinical strains of uropathogenic *E. coli* (two different clinical isolates labeled as strain S1 and strain S2 were tested; Fig. 4B and C)—unlabeled CSA-13 was equally effective in eradication of bacteria and the strongest bactericidal effect was observed 8–12 h after CSA-13 administration.

The beneficial activity of CSA-13 in vivo was also confirmed by the data collected from the histopathological analysis (Fig. 4D). Hematoxylin–eosin staining of tissue sections provide a general view of host responses to infection by assessing inflammation of infected bladder tissue, such as tissue edema, exfoliation of transitional epithelial cells, invasion of inflammatory cells in the mucosa, fibrosis of the lamina propria, and bladder mucosa hyperplasia (Fig. 4, Panel D 4-9). Histological display of the bladders collected from CSA-13-IRDye800CW treated mice (24 h after induction of infection and another 24 h after treatment) appeared free of inflammation and showed similar histology to bladder tissue collected from healthy control mice (Fig. 4, panel D 1-3; 10-12).

### Treatment with ceragenin CSA-13 prevents alterations of the mechanical properties of infected tissues

**Figure 5:**
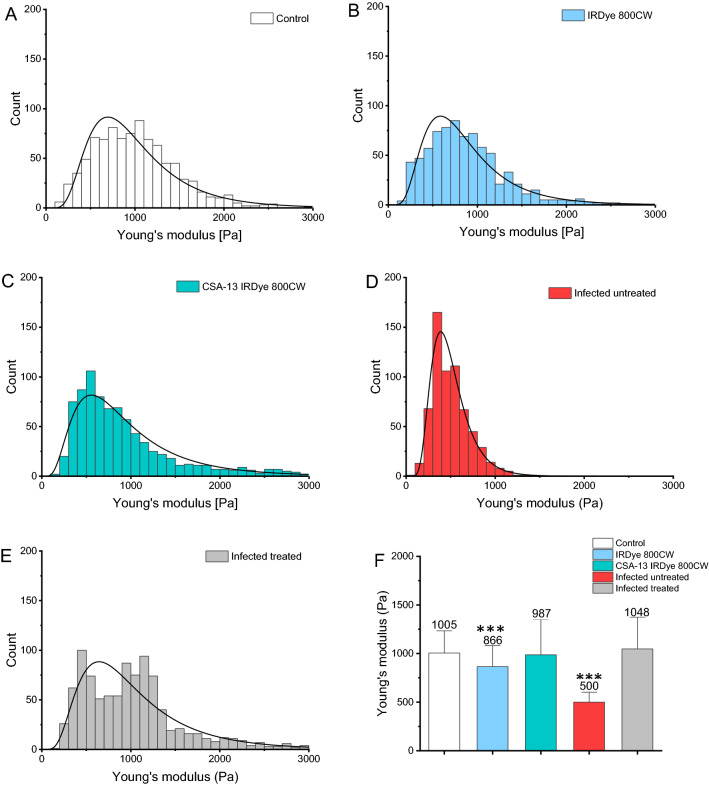
The Young's modulus values measured for each bladder samples collected from healthy animals, both untreated (**A**) and treated with IRDye800CW (**B**) or CSA-13-IRDye800CW (**C**), as well as *E. coli* Xen14-infected animals, both untreated (**D**) and treated (**E**) with CSA-13-IRDye800CW, with the logarithm of the normal distribution of the adjusted probability function density. The mean values of Young’s modulus ± standard deviation (**F**).

The analysis of the urinary bladder tissue by means of AFM (atomic force microscopy) made it possible to assess the effect of CSA-13 on tissue stiffness at the nanoscale. Figure 5 shows the distribution of Young's modulus (YM) values measured for each bladder tissue sample collected from healthy animals, both (1) untreated (n = 5; group 2) and (2) treated with IRDye800CW (n = 5, group 9) or (3) CSA-13-IRDye800CW (n = 5; group 8), as well as *E. coli Xen14* -infected animals, both (4) untreated (n = 5; group 2) and (5) treated with CSA-13-IRDye800CW (n = 5; group 3), with the log-normal distribution function fitted. The mean Young's modulus value of the control sample is equal to 1005 Pa, and IRDye800CW-treated tissues present only slightly lower stiffness what proofs that dye administration does not significantly interfere with tissue stiffness (Fig. 5A and B). Bladder tissues infected with *E. coli* Xen14 decreased in stiffness by 50% (YM = 500 ± 205 Pa) confirming that the microbiota is a potent modulator of the bladder tissue mechanical properties (Fig. 5D). Importantly, bladder tissues collected from infected mice that was treated with CSA-13-IRDye800CW exhibits mean stiffness similar to normal, untreated tissue (YM = 1048 ± 654 Pa) suggesting the presence of stiffness recovery mechanism after bacteria eradication (Fig. 5E). These results show that not only bacterial infection can modulate bladder tissue stiffness but also that bacteria eradication using CSA-13 can effectively reverse this process.

## Discussion

Currently, trimethoprim, β-lactams, fluoroquinolones, nitrofurantoin, and fosfomycin are considered as the first-line treatment options for acute uncomplicated cystitis; however, increased resistance to currently used antibiotics considerably limit their therapeutic efficiency^27^. In addition, many side effects are increasingly being reported with the above therapeutics. Particularly, fluoroquinolones are characterized by significant toxicity manifested by gastrointestinal disorders and connective tissue inflammation and similarly, combination of trimethoprim and sulfamethoxazole crosses readily the blood–brain barrier resulting potentially in adverse neurological effects^28,29^. As such, numerous cases of aseptic meningitis have been reported as a consequence involving high doses of trimethoprim alone or trimethoprim with sulfamethoxazole^28^. These observations serve as motivation for the quest for new therapeutic options for empiric therapy of UTIs. Here, we focus on the examination of antibacterial efficiency of ceragenins, and aim to explore their potential utility in the treatment of UTIs. Antimicrobial activity of ceragenins depends on their non-specific physicochemical properties such as amphipathic nature, and positive charge^30^ and their unique pharmacodynamic and pharmacokinetic features offer the opportunity to employ them in the treatment of bacteria-induced medical conditions. Our previous studies, demonstrating the (1) effectiveness of ceragenins, especially ceragenin CSA-13, against uropathogens^7^, as well as those reporting (2) high effectiveness against drug-resistant isolates of bacteria with the low tendency to induce drug-resistance^12,31^, motivate the current study. In addition, it is noteworthy that CSA-13 has been proven to be excreted with urine^18^. Potent bactericidal activity of ceragenins against *E. coli* Xen14 strains was confirmed using chemiluminescence measurement method and killing assay (Fig. 1), and those results are in agreement with our previous report^7^. Other publications also clearly show the effective action of CSAs against *E. coli* strains^17,32,33^. Importantly, CSA-13 maintains its activity also in the presence of urine (Table 2).

In one of the previous studies, Mitchell et al.^32^ presented that ceragenins have a distinctive mode of action and they propose a model in which ceragenins cross the outer layers of Gram-negative bacterial envelope and disrupt the inner membrane. Because the bacterial membrane and its phospholipid composition is a similar structural feature between Gram-positive and Gram-negative bacteria, and because resistance to drugs that target the membrane will require significant changes in membrane structure and composition, the membrane is an intriguing target for compounds such as ceragenins^34^. We confirmed that treatment of bacteria with ceragenins, particularly CSA-13, promotes the overproduction of reactive oxygen species leading to excessive oxidative stress in bacterial cells. Given that ROS can cause a variety of toxic effects, including DNA and RNA damage, membrane depolarization, protein carbonylation, and lipid peroxidation, leading directly to death of bacteria cell^12^, such ceragenin-mediated promotion of ROS formation is highly favorable event that may explain their bactericidal activity. Notably, such ceragenin-mediated ROS generation is dependent on the ceragenin used, since CSA-44 caused only a twofold increase of ROS production (at a concentration of 10 µg/mL), while CSA-13 and CSA-131 caused > 7 and > 6-fold increases, respectively, compared to control. Based on these findings, the antibacterial activity of CSA-13, CSA-44, and CSA-131 against bacteria is thought to include destruction of the inner and outer bacterial membranes, partially resulting from the induction of oxidative stress via the generation of ROS.

In addition to strong adherence to the bladder epithelium, *E. coli* are able to invade epithelial cells and form biofilm^35^. Biofilm development significantly impedes UTI treatment by shielding encapsulated bacteria from both the host immune response and antibiotic treatments. Moreover, bacteria embedded in a biofilm are in close contact, allowing for exchange of genetic material such as antibiotic resistance plasmids and transposons. For this reason, biofilm-producing bacteria are characterized by a spectrum of different phenotypes in terms of growth rate and gene transcription^36^. Biofilm formation in *E. coli* enhances colonization and increases the reoccurrence rate of UTIs, which can be difficult to treat due to antibiotic resistance. Importantly, the incidence of biofilm formation among uropathogenic *E. coli* can reach 70%^37^. For this reason, an additional experiment was performed, in which mature, 72 h biofilm formed by two different clinical strains of uropathogenic *E. coli* (S1 and S2) was subjected to 1 h treatment with varied concentrations of CSA-13 and CSA-131. Exposure of mature biofilm to CSAs results in a significant decrease of biofilm viability, which would be favorable in clinical settings due to limitation of biofilm-associated reinfections (data not shown). In addition, the ability of antibiotics from various classes to permeate the biofilm matrix and reach bacterial cells also varies. Clarithromycin with vancomycin or roxithromycin with imipenem are the most effective antibiofilm combinations mentioned in the literature^38^. According to our collected data, both in this and our previous reports^7^, ceragenins display strong ability to prevent biofilm formation by *E. coli* Xen14 and UPEC strains (Fig. 1D–F). CSA-13 and CSA-131 showed higher activity against biofilm in comparison to CSA-44.

A clinically-relevant issue is also the formation of biofilm on urethral stents and catheters, resulting in catheter-associated UTIs (CAUTIs) becoming one of the most prevalent care-associated illnesses worldwide^39^. Environmental conditions produced on the catheter surface make it a suitable place for bacterial adhesion and biofilm development^40^. For this reason, coating catheters with antimicrobials agents to make their surface less prone to pathogens attachment and biofilm development has been extensively studied^41^. Available data demonstrate that ceragenins can be used in this manner and appears to be well adapted for delivering medical devices with innate immune-like activity^42^. For instance, CSA-13 was used to create prototype lock solutions that were evaluated against biofilms and its anti-biofilm activity was comparable to ciprofloxacin-containing solution^42^. Given the mode of action of CSA-13 and the low likelihood of resistance, catheter lock solutions containing this ceragenin appear to offer an appealing, long-term strategy to eliminating biofilms in hemodialysis catheters^42^. In addition to potent antibacterial activity against planktonic and biofilm forms of bacteria, the ceragenins showed satisfactory biocompatibility tested using T24 bladder cells (Fig. 2A). This study revealed that more than 70%, 90% and 80% of T24 bladder cells survived at 10 μg/mL of CSA-13, CSA-44 and CSA-131, respectively (Fig. 2A). Importantly, ceragenins protected cells from bacteria-induced cell death (Fig. 2B and C). This effect seems to be primary governed by antimicrobial activities of CSAs themselves, but is highly favorable given the possible tissue damage resulting from excessive amount of bacteria present in urinary tract^43^.

Understanding drug absorption, distribution, metabolism, and elimination is an essential part of preclinical drug research, since it helps to identify leading drug candidates and potential first-in-human dosages. Therefore, in the first step of the animal study, we examined ceragenin distribution and accumulation in animal organs at 4, 8, 12 and 24 h after administration (Fig. 3B). As demonstrated, CSA-13 accumulates in the bladder in the first 4 h, whereas after 8 h it is also detected in the liver. After 24 h, the fluorescence intensity appeared to be derived from labeled ceragenin decay, which may indicate the complete elimination of this compound within the 24 h. This would be in agreement with previous studies suggesting that ceragenin accumulates in the kidney, among other areas, which further supports our validity in the use of ceragenin in UTI^44^. These results were confirmed by analysis of animal excreta (Figs. 3C and 4D). Given the relevance of urine ceragenin concentrations in the treatment of urinary tract infections and the kidneys' involvement in CSA-13 clearance, it would be highly valuable to investigate the influence of urinary pH on the pharmacokinetic and antibacterial activities of ceragenins. Although more thorough analyzes are required, it seems to us that ceragenin may be very similar in pharmacokinetic parameters to fluoroquinolones^18^. It is well-recognized that fluoroquinolones are largely eliminated from the human body through hepatic metabolism and renal excretion. The resulting consequence is that bile and urine end up with high active drug concentrations^45^. For instance, ciprofloxacin is rapidly excreted from the body under normal conditions, with an elimination half-life of 3–5 h^46^. Notably, fluroquinolones are excreted mostly in unchanged form. More carefully investigations are required to assess whether similar characteristic might be recognized for ceragenins.

Because of its capacity to identify the etiological factor and its antimicrobial sensitivities, urine culture has become the gold standard diagnostic test, allowing effective antibiotic stewardship^47^. This is especially beneficial in complex and recurring UTIs when successful bacterial eradication is required to avoid harmful consequences (e.g., upper urinary tract damage). Bacterial loads at various times post-infection can be used to monitor infections in the urinary tract quantitatively. The key result that supports the validity of ceragenin in the treatment of urinary infections are results from urine culture (Fig. 4A–C). We observed that the colonies grown from urine samples is significantly lower for ceragenin-treated animals, which reflects the therapeutic efficiency of CSA-13 in this animal model. To date, the in vivo use of ceragenin has been shown to have great potential in fighting bacterial, fungal and viral infections^15,18^. Notably ceragenins are not peptide based and are not degraded by human protease, so they have a prolonged tissue half-life^48^. Moreover, ceragenins were noted to be highly resistant to inhibitory effects of environmental factors at the infection site, which makes them more appealing as antimicrobial agents than endogenous antimicrobial peptides^49^. Undoubtedly, the above features, but also very good pharmacokinetic parameters, inspire exploration for new properties of ceragenin to be used with other infections.

Nanoindentation techniques such as atomic force microscopy, whose capabilities enable the characterization of the advancement of pathological diseases, has recently been identified. The assessment of elasticity modulus (Young`s modulus) can be utilized to explain mechanical basis of pathophysiology development by quantifying local cell or tissue stiffness^50^. In an increasing number of in vitro studies, particularly those focused on cancer, mechanical characterization has been conducted on pathologically altered specimens to compare their properties with normal counterparts, and demonstrate significant softening of malignant cells^51–53^. In our study, we focused on a direct comparison of mechanical properties between bladder tissues collected from healthy animals, those untreated, subjected to bacterial challenge and treated with CSA-13-IRDye800CW. The values of Young's modulus for healthy and infected bladder tissues were found to be substantially different and significantly lower post *E. coli* Xen14 infection. This finding is in agreement with previous reports showing that pathological dysbiosis mediates host ECM degradation due to bacterial secretion of elastases, hyaluronidases, collagenases and alkaline phosphatases^54–57^. Interestingly, ceragenin CSA-13 treatment reversed mechanical softening of the infected tissues, thus confirming that effective bacteria eradication allows to restore physiological homeostasis. This observation is also in agreement with previous reports on *Listeria monocytogenes* or *Helicobacter pylori* infections of cells^58,59^. Most recently, we have observed a similar tendency in *Helicobacter pylori*-positive stomach tissues collected from pediatric patients, as well as when examining mechanical response of human gastric cells to heat-inactivated bacteria using in vitro settings. At the cellular level, the decrease of cellular and tissue stiffness was concluded as associated with tuning properties of gastric cells to bacteria exposure and remodeling of cell cytoskeleton. Importantly, such dynamic nanomechanical changes at the level of single cells and tissues might hypothetically affect intracellular molecular processes leading ultimately to neoplastic transformation^59^. Possibly, such effects might occur also in our experimental settings and reversal of the tendency in stiffnesses of untreated and treated tissue samples reflects the prevention of bacteria-associated tissue damage by tested ceragenins and for a long-term prevention of UTI associated bladder cancers. Although inflammatory parameters in UTI-suffering mice were not examined in this study, it should be also assumed that observed stiffness effects (increase of cellular stiffness upon treatment) might be also associated with anti-inflammatory activities of ceragenins, similarly as recorded for gelsolin-derived peptides in keratinocyte-based model of skin infection^60^. Potentially, the similar phenomena occurs in in vivo settings, leading to improvement of clinical outcomes of treated animals. Moreover, we would like to note that histopathological analysis of bladder samples collected from treated animals confirmed that the markers of inflammation (tissue edema, exfoliation of transitional epithelial cells, invasion of inflammatory cells in the mucosa, fibrosis of the lamina propria, and bladder mucosa hyperplasia) were considerably decreased in ceragenin-treated animals when compared to control ones. Moreover, the ability of ceragenin to diminish inflammation induced by pathological process was also presented by our research team when analyzing the anti-cancer activities of CSA-131 and its nanosystem^61^. A compelling body of evidence confirms also that the elastic modulus is an important factor influencing bacterial attachment, growth and biofilm formation, suggesting that the stiffening of tissues upon ceragenin administration in UTI-suffering animals creates an environment unfavorable for bacteria replication and virulency. In one of the studies, Song et al. reported that the ability of *E. coli* to adhere and to form biofilms on hydrogel is decreased when stiffness increases^62^. Similarly, a decrease in the surface stiffness was reported to promote both the growth and the attachment of *E. coli* and *P. aeruginosa* cells^63,64^. The stiffness of bladder epithelial cells was reported to regulate endosomal escape and intracellular proliferation of uropathogenic *E. coli* in the cytoplasm of bladder epithelial cells via a Rho GTPase Rho B-dependent mechanism^65^. The sum of these reports clearly suggests that CSA-13 may display beneficial effects in urinary tract infection by both direct (i.e. bactericidal effect) and indirect mechanisms, promoting the formation of microenvironment with the biophysical features unfavorable for bacterial spreading and replication.

## Conclusion

The set of advantageous parameters of ceragenins, including a broad spectrum of antimicrobial activity, maintenance of its activity in the presence of urine, low toxicity and excretion by kidneys, as well as promotion of favorable nanomechanical features of bladder tissues makes it an worth-noting molecule to develop in the context of new antibiotic to treat UTIs.

## Materials and methods

### Bacterial strains

The research was performed using bioluminescent *E. coli* Xen 14 strain (PerkinElmer Inc., Waltham, MA, United States), and two clinical strains of *E. coli* isolated from patients (women, age 27 and 36) hospitalized in Independent Public Province Hospital of Jan Sniadecki in Białystok that were diagnosed with cystisis, before antibiotic therapy. The *E. coli* strains were isolated from clean midstream urine, and were denoted here as strain S1 (S1) and strain S2 (S2). Details on clinical symptoms, a summary of therapy, and other pertinent data were acquired from the hospital information system and laboratory results.

### Evaluated compounds

CSA-13, CSA-44 and CSA-131 were synthesized from cholic acid as previously described^66^. For the purpose of experiments using the animal model, CSA-13 was conjugated with IRDye 800CW fluorescent probe (CSA-13-IRDye 800CW). For this purpose, ceragenin was mixed with IRDye 800CW NHS ester (Li-COR Biosenses, Lincoln, Nebraska, USA) in 10:1 ratio. The mixture was incubated overnight at 4 ºC allowing bond formation between free –NH_2_ groups of CSA-13 and -NHS reactive group of the probe.

### In vitro antimicrobial activity testing

The minimal inhibitory (MIC) and minimal bactericidal (MBC) concentrations of tested ceragenins (CSA-13, CSA-44, CSA-131) against *E. coli* Xen14 and two clinical strains of uropathogenic *E. coli* (S1 and S2) were determined in LB (Luria–Bertani) broth using the microdilution method with agent concentrations ranging from 0.5 to 16 µg/mL. The MIC for a given titration series was the lowest ceragenin concentration yielding no visible growth after overnight culture. For MBC determination, aliquots from each overnight MIC dilution series underwent quantitative plating on agar. The MBC was the lowest ceragenins concentration that yielded a ≥ 99.9% decrease in viable counts. The same procedure was employed for estimation of MICs and MBCs of CSA-13 and CSA-13-IRDye800CW in the present of human urine and blood plasma. Antibacterial activity of ceragenins against *E. coli* Xen14 was confirmed quantitatively using chemiluminescence intensity measurements (allowing investigation of the metabolic activity of ceragenin-treated bacteria) and colony counting assays^67,68^. For the purpose of chemiluminescence measurements, *E. coli* Xen14 colonies were grown to mid-log phase at 37 °C, resuspended in Luria–Bertani broth, and diluted to 10^9^ CFU/mL. Then, 100 μL of bacteria suspension was added to each well containing 10 μg/mL of tested compounds. Chemiluminescence intensity was measured during 1 h using a Varioskan Lux microplate reader (Thermo Fisher Scientific, USA). Colonies forming abilities of treated *E. coli* cells were explored upon incubation with tested agents at concentrations ranging from 1 to 50 µg/mL. Next, the plates were put on ice, and the suspensions were diluted 10- to 1000-fold in PBS. The CFUs were then determined by plating 10 μL aliquots on LB agar for overnight culture at 37 °C. After being exposed to the tested agent, cell survival was expressed in log CFU.

### Generation of reactive oxygen species (ROS)

The fluorescent probe 2′,7′-dichlorofluorescein diacetate (DCFH-DA, Sigma-Aldrich, USA) was used to estimate the generation of ROS induced by ceragenins^69^. Bacterial cell suspensions (OD_600_ of 0.1) were pipetted into 96-well black plates. Then, CSA-13, CSA-44, and CSA-131 were added to each well in concentrations ranging from 0 to 100 μg/mL. Later, 20 μM DFCH-DA in PBS was prepared, mixed and added, followed by incubation for 60 min. At excitation/emission wavelengths of 488/535 nm, fluorescence was measured using the microplate reader Varioskan Lux.

### Anti-biofilm activity

An incubation of *E. coli* Xen14 with different concentrations (2–100 μg/mL) of tested compounds for 24–72 h at 37 °C was performed to determine the ability of ceragenins to prevent biofilm formation. An overnight pathogen culture was diluted to 10^5^ CFU/mL. In 96-well polystyrene plates, bacterial suspensions and analyzed agents were mounted, and a biofilm was allowed to expand. The growth medium containing planktonic bacteria was removed after incubation, and the wells were washed 3× with PBS. Crystal violet, 0.1% (w/v), was used to stain the biofilm. The crystal violet working solution was removed after 15 min, and the plates were thoroughly rinsed. The residual biofilm-staining crystal violet was dissolved in 95% ethanol to assess the mass of biofilm. Spectrophotometry analysis (λ = 580 nm) was used to determine the amount of extracted stain.

### Cell culture

Human urinary bladder cancer cells T24 (HTB-4™, ATCC) were maintained in McCoy’s 5A cell culture medium supplemented with 10% fetal bovine serum, 50 U/ml penicillin, and 50 mg/mL streptomycin at 37 °C in a 5% CO_2_ incubator.

### Cytotoxicity assays in bacteria-free and bacteria-containing conditions

To assess the cytotoxicity of tested ceragenins toward human urinary bladder cancer cells, a previously established protocol was employed^70,71^. Briefly, T24 cells were seeded in a 48-well plate at a density of 15–20 × 10^3^ per well and treated with different concentrations of ceragenins for 24 h. The cells were washed with PBS and incubated in a solution of methylthiazoletetrazole (MTT) at a final concentration of 0.5 mg/mL for 4 h. Measurement of spectrophotometric absorbance at 550 nm was performed using a Varioskan Lux microplate reader. Untreated cells were considered to be 100% viable. Each measurement was repeated at least 3 times. An analogous procedure was employed to investigate the abilities of ceragenins to prevent bacteria-induced cytotoxicity. In this experimental setting, *E. coli* Xen14 strain, both freshly inoculated or heat-inactivated (by boiling bacterial suspension at 121 ºC for 15 min; efficiency of bacteria killing was confirmed by plating the inactivated suspension on agar plates to confirm the loss of outgrowth ability), were suspended in cell culture medium (with the final inoculum being equivalent to 10^6^ CFU/mL) and cultured with ceragenins at doses ranging from 0 to 10 µg/mL.

### Compounds used for the animal model

Among the ceragenins, CSA-13 displayed the most favorable activity against *E. coli* Xen14 and clinical uropathogenic isolates of bacteria (S1 and S2) and was selected for testing in the animal study. Considering the lack of differences in antimicrobial activity of CSA-13 and CSA-13-IRDye800CW and the technical requirements for following biodistribution analyses, we decided to use fluorescent-labelled CSA-13 as a therapeutic agent for animal groups inoculated with *E. coli* Xen14 strain (group 5). At the same time, to ensure that conjunction of CSA-13 with fluorescent probe did not affect urinary excretion of CSA-13, animals inoculated with clinical strains of uropathogenic *E. coli* (S1 and S2, groups 6–9) were treated with unlabeled ceragenin (groups 7, 9). Experimental groups used for animal study are indicated in Table 3.

**Table 3:** Groups of animals used in animal study.

Group	Purpose of experiment	Number of animals	UTI induction/used strain	Administrated compound	Dose of administrated compound	Urine culture	Route of drug administration
1	Control	5	–	0,9% sterile saline	0.1 mL	+	Intravenously
2	Antibacterial efficiency	5	*E. coli* Xen14	0,9% sterile saline	0.1 mL	+	
3		5	*E. coli* Xen14	CSA-13-IRDye 800CW	10 µg/mL (including 1 µg/mL of IRDye® 800CW)	+	
4		5	*E. coli* clinical strain (S1)	0,9% sterile saline	0.1 mL	+	
5		5	*E. coli* clinical strain (S1)	CSA-13	10 µg/mL	+	
6		5	*E. coli* clinical strain (S2)	0,9% sterile saline	0.1 mL	+	
7		5	*E. coli* clinical strain (S2)	CSA-13	10 µg/mL	+	
8	Biodistibution assesment	5	–	CSA-13-IRDye 800CW	10 µg/mL (including 1 µg/mL of IRDye® 800CW)	−	Intravenously
9		5	–	IRDye 800CW	1 µg/mL	−	

Urinary tract infection (Day 0) induction of urinary tract infection by *E. coli* Xen14 and two clinical strain of *E. coli* (S1 and S2) administration (n = 5 per group; group 2–7). (Day 1) 24 h after induction, mice were divided into 2 groups: (1) UTI-suffering, untreated animals that obtained intravenously 0.1 mL of 0.9% NaCl (n = 5; group 2, 4, 6), and (2) UTI-suffering animals, treated *i.v.* with CSA-13-IRDye800CW or CSA-13 at dosage of 5 µg/g of body weight (n = 5 per each group; group 3,5,7). (Day 2) the urine was collected from the animals from 1 to 7 groups at the specified times (0 h, 4 h, 8 h, 12 h and 24 h after treatment) and then were plated on MacConkey agar plates to determine bacterial titers.

Part 2: Biodistribution of CSA-13. (Day 0) Mice were injected with 0.1 mL of 0.9% NaCl (n = 15). (Day 1) mice were divided into the following groups: Group 8 and 9 were injected intravenously with CSA-13-IRDye800CW (n = 5; group 8) or unmodified IRDye800CW dye (n = 5; group 9), respectively. (Day 2) Animals from 8 and 9 groups were imaged using the Pearl®Trilogy small animal imaging system (Li-COR, Lincoln, NB, USA) at different times after treatment with CSA-13-IRDye800CW (n = 5; group 8) or unmodified IRDye800CW dye (n = 5; group 9) (0 h, 4 h, 8 h, and 12 h), after that animals from all groups were sacrificed and tissues were analyzed.

### Induction of urinary tract infection in animals

Nude CBy.Cg-Foxn1<nu>/cmdb female mice (The Jackson Laboratory; Bar Harbor, ME USA), aged 9–12 weeks, were used. Mice body weight was monitored prior to induction of infection, after 8 and 12 h post treatment and before sacrificing. Before inducing UTI, urine samples from animals were obtained and cultured to confirm the lack of any bacteria presence in urine. Collected data showed that there were no bacteria in the urine, indicating that all mice subject to this study were free of bacteria prior to induction of infection. To induce infection, female mice weighing from 20 to 25 g were put on their backs after being anesthetized with 5% isoflurane gas and then an inoculum of bacteria [*E. coli* Xen14 (n = 5, groups 2–3), or 2 clinical strains of uropathogenic *E. coli* S1 and S2 (n = 5 per each group, groups 4–7)] in 50 μL phosphate-buffered saline (PBS; 1.2 × 10^7^ CFU) was administrated over 30–45 s directly into the bladder via urethral catheter^72^. Control mice (groups 1, 2, 4, 6) were injected with 0.1 mL of 0.9% NaCl. Following inoculation, the mouse bladders were drained for 4 h to ensure full *E. coli* adhesion with membrane receptors of bladder epithelial cells. The bacterial inoculum administered was consistent with previously published studies^73^. After bacterial challenge, the appropriate induction of urinary infection was confirmed by both culture of collected urine (bacteriuria) and histopathological evaluation of the urinary bladder (tissue edema, exfoliation of transitional epithelial cells, invasion of inflammatory cells in the mucosa, bladder mucosa hyperplasia), as well as changes in blood parameters (WBC, RBC, PLT)—Supplementary Table 1.

### Treatment procedure

Female mice, 24 h after bacterial challenge with *E. coli* Xen14, were divided into 2 groups: (1) UTI-suffering, untreated animals, which were dosed intravenously with 0.1 mL of 0.9% NaCl (n = 2; group 4), and (2) UTI-suffering animals, treated *i.v.* with CSA-13-IRDye800CW at a dose of 5 µg/g of body weight (n = 5; group 3). As a negative control, (3) healthy animals (not challenged with *E. coli* suspension) were treated with 0.9% NaCl (n = 5; group 1). Another group of mice, 24 h after bacterial challenge (*E. coli* clinical strains, S1 and S2), were divided into 2 groups: (1) UTI-suffering, untreated animals, which obtained intravenously 0.1 mL of 0.9% NaCl (2 strains of clinical strains of *E. coli*, S1 and S2, 5 mice per group; groups 4 and 6), and (2) UTI-suffering animals, treated *i.v.* with CSA-13 at dosage of 5 µg/g of body weight (2 strains of clinical strains of *E. coli*, S1 and S2, 5 mice per group; groups 5 and 7). As a negative control, (3) healthy animals (not challenged with *E. coli* suspension) were treated with 0.9% NaCl (n = 5; group 1).

### Assessment of bacterial presence in urine

Urine was collected from the animals of groups 1–7 at the specified times (0, 4, 8, 12 and 24 h after treatment) by gentle compression of the bladder through the abdominal wall. A micropipette was used to extract urine samples (5 µL) from the external urethral meatus. Serial dilutions of urine samples were plated on MacConkey agar plates to determine bacterial counts.

### Biodistribution of CSA-13-IRDye800CW

For the purpose of analysis of biodistribution of ceragenin, uninfected animals from groups 8 and 9 were injected intravenously with CSA-13-IRDye800CW (n = 5) or unmodified IRDye800CW dye (n = 5), respectively. Mice were anesthetized with 3% isoflurane gas prior to imaging, and the entire animal was imaged for a period of 2 min using the Pearl®Trilogy small animal imaging system (Li-COR, Lincoln, NB, USA) at different times after treatment (0, 4, 8, and 12 h). Mice were held anesthetized with 1–2% isoflurane during the imaging process. Using the Image Studio program 5.2, total photon emissions from specified regions of interest within each mouse image were quantified. The photon signals in ventral images of each mouse were quantified. Mice were euthanized during the procedure and after the final imaging time point, and organs were harvested and scanned using the 800 nm channel for evaluation of the fluorescent location and intensity of CSA-13IRDye800CW/IRDye800CW. Additionally, a portion of collected urine and feces were placed on a glass slide and scanned to determine the intensity of the signal derived from CSA-13-IRDye800CW (n = 5; group 8) or unmodified IRDye800CW dye (n = 5; group 9), respectively.

### Histopathological analysis

Bladder tissue samples were surgically removed upon sacrificing animals from groups 1–3, sliced and embedded in 4% buffered formalin. Subsequently, sections were stained with hematoxylin–eosin and histopathological analysis was performed.

### Atomic force microscopy

Increasing number of studies indicate that nanoscale mechanical properties of biological tissues, such as stiffness, might be used as a marker of pathologic state^59^. For the purpose of nanomechanical properties evaluation, fragments of bladder tissues collected from animals inoculated with *E. coli* Xen14 strain (groups 1, 2, 3, 8 and 9) were stored in Tissue Storage Solution (MACS Media Bergisch Gladbach Germany) and measured within two hours post-surgery. Control, infected and treated bladder tissues were evaluated using atomic force microscope (NanoWizard 4 BioScience JPK Instruments Bruker, Berlin, Germany) working in the Force Spectroscopy mode. Force indentation curves were collected using a silicon nitride cantilevers with a nominal spring constant of 0.7 N/m and measured spring constant in the range of 0.7–1.0 N/m (as determined using thermal tune method), with a 4.5 μm diameter polystyrene bead attached. The AFM cantilevers were manufactured by Novascan Technologies, Inc. (Boone, USA). Tissues were glued onto a Petri dish and immersed in DMEM for the whole measurements that were performed at room temperature. Up to 15 indentation maps consisting of 8 × 8 points corresponding to a scan area of 10 × 10 µm were collected from multiple random places for each tissue sample. Experiments were repeated using five samples per every group studied. The difference between the cantilever deflection on a rigid Petri dish surface and compliant tissue sample describes the deformation of the tissue under the external load. By plotting the force used for sample deformation against the depth of cantilever`s indentation, force-versus-indentation curves were obtained. To determine the elastic modulus (so-called Young’s modulus) that represents sample stiffness, curves were fitted using Hertz contact model as described elsewhere^74^. Young's modulus (YM) values distributions for each group were prepared, and the mean YM values for all healthy and infected tissues and cells along with standard deviations were calculated.

### Ethical statement

All methods were performed in accordance with the relevant guidelines and regulations. Clinical strains of *E. coli* isolated from the patients with urinary tract infections were acquired under the approval of Bioethics Committee at the Jan Kochanowski University in Kielce, Faculty of Medicine and Health Sciences (no. 22/2019). Human urine and blood plasma were collected upon approval of Bioethics Committee at the Jan Kochanowski University in Kielce, Faculty of Medicine and Health Sciences (no. 22/2019) and Bioethics Committee at the Medical University of Bialystok (no. R-I-002/231/2019), respectively. Research involving human research participants have been performed in accordance with the Declaration of Helsinki and informed consent has been obtained from the participants involved. Animal experiments were approved by the Local Ethic Committee in Olsztyn of the University of Warmia and Mazury no 64/2020. Animal study is reported in accordance with ARRIVE guidelines.

### Statistical analysis

The data described are results from three independent experiments ± SD. The significance of differences was determined using the two-tailed Student’s t-test. Statistical analyses were performed using OriginPro 2020 (OriginLab Corporation, Northampton, USA). P < 0.05 was considered to be statistically significant.

## Supplementary Information

Supplementary Information.

## Data Availability

The datasets generated during and/or analyzed during the current study are available from the corresponding author on reasonable request.
