# Supplementary material for: Ceragenin CSA-13 displays high antibacterial efficiency in a mouse model of urinary tract infection
Source: Sci Rep. 2022 Nov 10;12:19164. doi: 10.1038/s41598-022-23281-y (PMC9649698; doi:10.1038/s41598-022-23281-y)
Supplement: Supplementary file 1 — Supplementary Information. [file 41598_2022_23281_MOESM1_ESM.pdf]

Supplementary Table 1. Morphological and biochemical parameters of blood collected from mice used in this study.

	WBC	RBC	HGB	HCT	MCV	MCH	MCHC	PLT
**Control**	6,4	9,0	14,4	53,2	58,6	15,9	27,2	298,9
**Untreated**	8,2	9,8	15,9	57,9	58,8	16,2	27,5	324,1
**Treated**	7,8	9,0	14,9	53,2	58,8	16,1	27,2	316,5
**CSA-13 IRDye800CW**	5,5	9,0	14,5	52,3	58,4	16,1	27,7	354,4
**IRDye800CW**	6,0	7,3	11,8	42,6	57,8	16,1	27,9	209,4

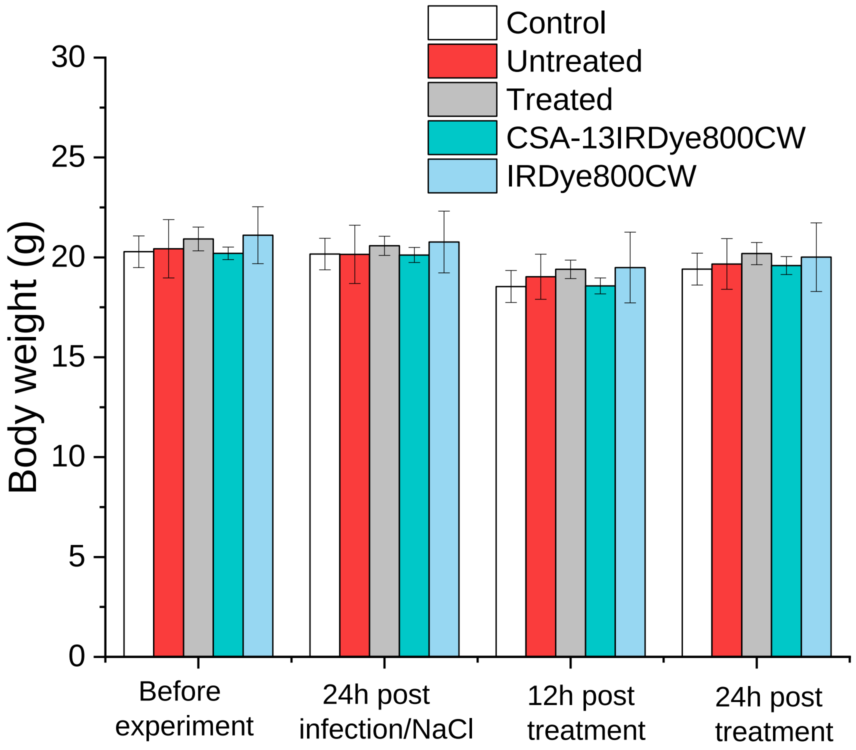

Supplementary Figure 1. Body weight of animals used in this study measured before experiment, 24h post infection and 12 and 24 hours post treatment.
